# How safe and effective are flow diverters for the treatment of unruptured small/medium intracranial aneurysms of the internal carotid artery? Meta-analysis for evidence-based performance goals

**DOI:** 10.1136/neurintsurg-2019-015535

**Published:** 2020-01-31

**Authors:** David Fiorella, Larry Gache, Diana Frame, Adam S Arthur

**Affiliations:** 1 Stony Brook University Hospital, Stony Brook, New York, USA; 2 CTI Clinical Trial and Consulting Services, Covington, Kentucky, USA; 3 Neurosurgery, University of Tennessee Health Science Center, Memphis, Tennessee, USA; 4 Semmes-Murphey Clinic, Memphis, Tennessee, USA

**Keywords:** intracranial aneurysm, flow diverters, endovascular therapy

## Abstract

**Background:**

The objective of this meta-analysis was to establish safety and effectiveness benchmarks for endovascular therapy of unruptured small-to-medium internal carotid artery (ICA) aneurysms using flow diverters.

**Methods:**

A systematic literature review and subsequent meta-analysis were performed using best research methods. Studies of any design with at least 10 patients treated with flow diverters for predominantly (≥90%) unruptured small/medium ICA aneurysms and ≥6 month follow-up were included. The primary effectiveness endpoint was complete aneurysm occlusion rate at 12 months. The primary safety endpoint was a composite measure of cumulative events that could indicate a stroke or neurologic death: any death, stroke, intracranial hemorrhage, or worsening on the modified Rankin Scale.

**Results:**

41 studies (2614 patients) met eligibility criteria for the meta-analysis. The core lab adjusted complete occlusion rate was 74.9% (95% CI 69.6% to 79.8%) at 12 months for studies using any flow diverter. With an aim of generating performance goals for a US Investigational Device Exemption (IDE) study, a pre-specified analysis was conducted using only studies with flow diverters commercially available in the USA. In this cohort, 12 month complete occlusion was 74.6% (95% CI 66.8% to 81.7%). The primary safety event rate for flow diverters commercially available in the USA was 7.8% (95% CI 4.8% to 11.4%).

**Conclusions:**

The treatment of small and medium-sized aneurysms with flow diverters is effective in achieving curative reconstruction in most cases and is associated with low rates of morbidity and mortality. This meta-analysis informs robust performance goals for evaluating new flow diverters in small/medium unruptured carotid aneurysms.

## Introduction

Flow diverters are used to provide a curative endovascular treatment option for wide-necked side-wall aneurysms.^1–4^ Most of the available high-quality data for these devices have been derived from the treatment of large and giant aneurysms. The effectiveness and safety of flow diversion for the treatment of these lesions have been well characterized. In clinical practice, the majority of aneurysms treated with flow diverters are actually small and medium-sized aneurysms. The objective of the present systematic literature review and meta-analysis was to establish safety and effectiveness benchmarks for flow diversion of predominantly unruptured small-to-medium internal carotid artery (ICA) aneurysms.

## Methods

A systematic literature review and meta-analysis were performed using best research methods in compliance with Preferred Reporting Items for Systematic Reviews and Meta-Analyses (PRISMA) guidelines.^5^ A protocol and statistical analysis plan were prepared before conducting the search and meta-analysis, respectively, in order to minimize bias. The systematic review protocol was registered in the International Prospective Register of Systematic Reviews (PROSPERO).^6^

### Search strategy

Electronic searches were performed in PubMed/MEDLINE for English language articles published between January 1, 2008 and June 30, 2018, and supplemented by manual checks of the reference lists of recent reviews. In addition to the search of journal publications, relevant abstracts presented at the International Stroke Conference and the Society of NeuroInterventional Surgery Annual Meetings were sought for the years 2017 and 2018. Details of the search strategy are available in online supplement 1.

10.1136/neurintsurg-2019-015535.supp1 Supplementary data

### Study selection

Studies of any design, with at least 10 individuals treated with flow diverters for unruptured aneurysms (at least 90% of the study population with unruptured aneurysms), and with a reported follow-up duration of at least 6 months, were sought using the search methods above. In addition to the initial selection criteria, the following requirements were prospectively applied to focus the meta-analysis on the most relevant studies: (1) small/medium aneurysms: the reported average (mean or median) aneurysm size was 12 mm or smaller for the study population, or authors described all patients as having “small” or “small/medium” aneurysms; and (2) at least 90% of aneurysms were in the ICA. Studies with a mixed population of aneurysm locations, but with separately reported effectiveness and/or safety outcomes for the subgroup of patients with ICA aneurysms, were extracted for the ICA subgroup only. For studies included in the safety analyses, all components of the composite safety endpoint were required to ensure complete reporting.

In order to generate information most relevant to performance goals for a US Investigational Device Exemption (IDE) study, a pre-specified analysis was conducted using studies that employed only flow diverters commercially available in the USA (ie, the Pipeline Embolization Device (Medtronic, Fridley, MN) and Surpass flow diverter (Stryker, Kalamazoo, MI)). Publications reporting data for the same or overlapping patient population as another included study were excluded from the meta-analysis to avoid double-counting patients. A single researcher screened titles and abstracts of citations found in the search (level I screening), with questions resolved by a second reviewer. Potentially eligible citations were then obtained in full text and screened for fit with the study selection criteria (level II screening). At level II screening, two reviewers independently evaluated each study for eligibility, and the reason for exclusion was captured for all studies not included in the meta-analysis.

### Data extraction and definition of endpoints

Data extraction was conducted on all studies meeting meta-analysis eligibility criteria, with one reviewer extracting all study and patient characteristics and outcomes from the full text of the study, and a second reviewer checking each data element against the full text of the study (extraction and consensus process). All data extraction was conducted independently from the statistical analysis.

The primary effectiveness endpoint was complete aneurysm occlusion rate at 12 month follow-up, defined as 12±2 months (10–14 months). For studies that reported angiographic outcomes as read by the investigator (“self-reported” publications), a core laboratory correction factor of 12%, as defined by Fiorella *et al*, was used to estimate adjusted occlusion rates that could be compared with core lab-read or independently confirmed rates.^7^

Because safety events are rare and there is significant variability in reporting safety endpoints in the published literature, the primary safety endpoint used was a composite measure of cumulative events. These events represent outcomes that could indicate a stroke or neurologic death. Patients were classified as having the composite event if they experienced one or more of the following component events (periprocedural or during follow-up): death, stroke (hemorrhagic, ischemic, or unknown/not specified), intracranial hemorrhage, or worsening condition on the modified Rankin Scale (mRS).

### Meta-analysis

Analyses were performed with random-effects inverse variance weighting models, using both the DerSimonian-Laird (DL) and Hartung-Knapp-Sidik-Jonkman (HKSJ) methods. Compared with DL, the HKSJ method has been found to provide more consistently adequate error rates and is becoming increasingly accepted as a more appropriate method;^8^ therefore, HKSJ results were considered primary. Effect sizes were calculated as event rates using the arcsine transformation. Residual and influence checks, including the leave-one-out method and manual inspection for outlying and influential studies, were performed on all analyses. Sensitivity and subgroup analyses were employed to understand the robustness of the effect size estimates and to evaluate potential sources of heterogeneity. Publication bias and other systematic heterogeneity related to sample size was assessed using funnel plots and Egger’s regression test. Data manipulation and statistical analyses were performed using SAS (v.9.4, SAS Institute, Cary, NC) and R (meta package v.4.9.2, Foundation for Statistical Computing, Vienna, Austria).

## Results

**Figure 1:**
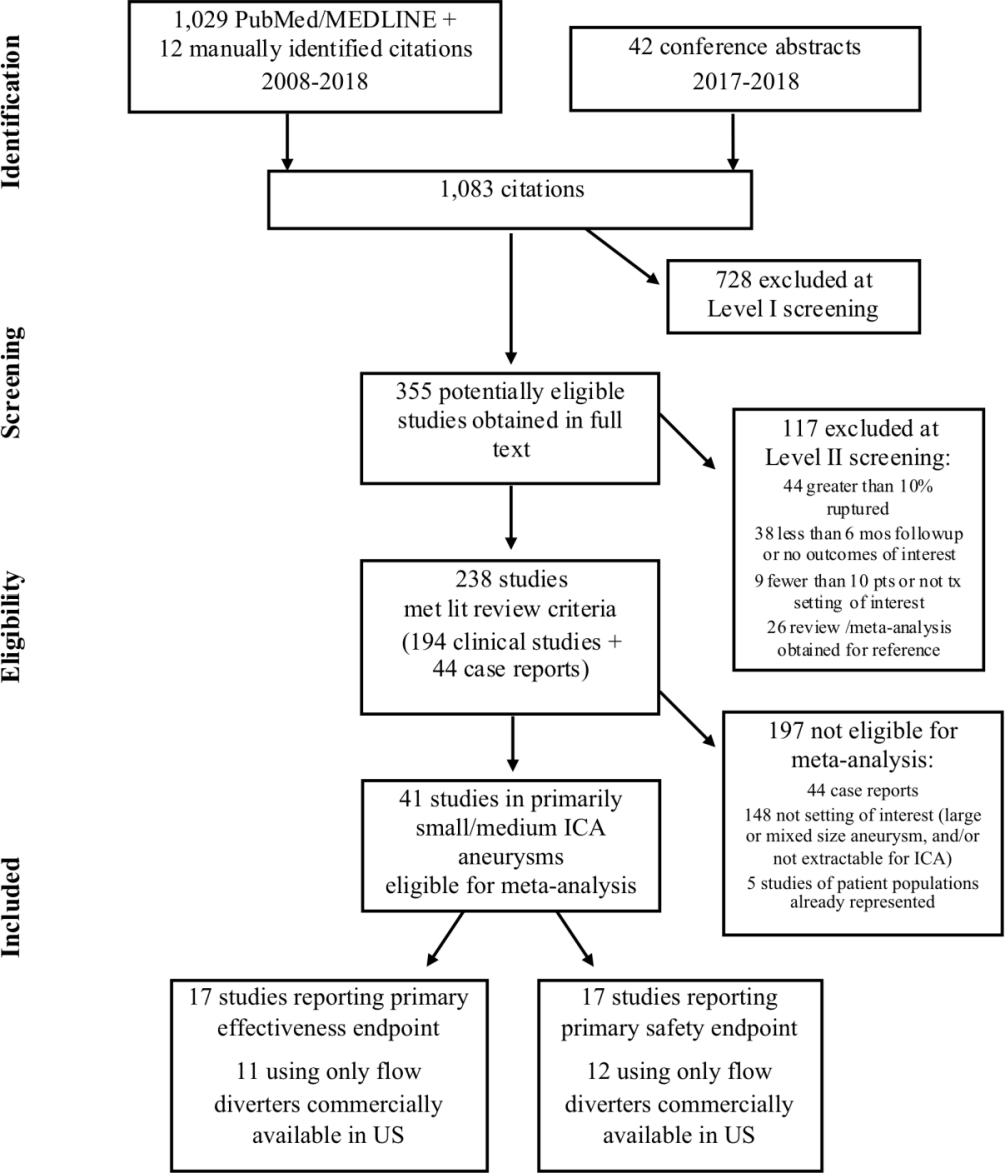
PRISMA flow diagram. ICA, internal carotid artery; PRISMA, Preferred Reporting Items for Systematic Reviews and Meta-Analyses.

The literature search yielded a total of 1083 citations, of which 41 studies met eligibility criteria for the literature review and meta-analysis (figure 1). These studies enrolled 2614 patients being treated for primarily unruptured small/medium aneurysms located in the ICA. Two studies were available only in abstract form.^9 10^ Most studies used Pipeline and were conducted in the USA or Europe (table 1). There were 35 case series (most retrospective) and six retrospective comparative studies. No randomized controlled trials were found satisfying the study selection criteria.

**Table 1:** Study characteristics

Study characteristic	Studies, n	Patients, n*
Article type		
Full	39	2452
Abstract	2	162
Study design		
Retrospective case series	28	1881
Prospective case series	7	428
Retrospective comparative study (non-randomized)	6	305
Flow-diverter device		
Pipeline†	29	1972
Surpass†	2	23
DERIVO	1	24
FRED	1	20
Pipeline Shield	1	50
Silk	1	246
Mixed	6	279
Method for occlusion verification		
Core lab/independent review	14	678
Site reported/not specified	27	1936
Method for adverse events verification		
Independent review/clinical events committee	8	857
Site reported/not specified	33	1757
Region		
North America	20	1230
Europe	13	452
Asia-Pacific	2	198
Latin America	2	362
Multi-continental	4	372
Percent unruptured category		
90–<95%	7	836
95–<100%	5	374
100%	25	1223
Not reported‡	4	181
Aneurysm size category		
Small: mean/median <7 mm or exclusively small size	16	1515
Medium: mean/median 7–12 mm	22	789
Mean/median not reported§	3	310
Percent ICA category		
90–<95%	10	413
95–<100%	4	545
100%	27	1656

*Two studies did not report number of eligible patients: 57 aneurysms in the subset of eligible patients^18^ and 118 aneurysms in the subset of eligible patients.^19^

†Commercially available in USA at the time the review was conducted.

‡Unruptured percentage determined to be at least 90% per selection criteria, but exact number not extractable.

§Mean/median aneurysm size determined to be ≤12 mm per selection criteria, but exact number not extractable.

ICA, internal carotid artery.

Eleven studies that reported complete occlusion rates at 12 months and employed only flow diverters commercially available in the USA were available for meta-analysis. The overall core lab adjusted rate of complete occlusion at 12 months was 74.6% (table 2). The lower bound of the 95% CI, pre-specified for performance goal usage, was 66.8%. When publications reporting use of all flow diverters were analyzed (ie, not limited to devices commercially available in the USA), the pooled estimate for core lab adjusted complete occlusion was not significantly different (74.9%). Both analyses found no evidence of publication bias or small-study effects (online supplement 2). Residual and pre-planned sensitivity analyses found the estimates to be robust.

10.1136/neurintsurg-2019-015535.supp2 Supplementary data

**Table 2:** Complete occlusion at 12 months

	Flow diverters available commercially in the USA	All flow diverters
Pooled estimate, %	74.6	74.9
95% CI	66.8 to 81.7	69.6 to 79.8
Studies, n	11	17

**Figure 2:**
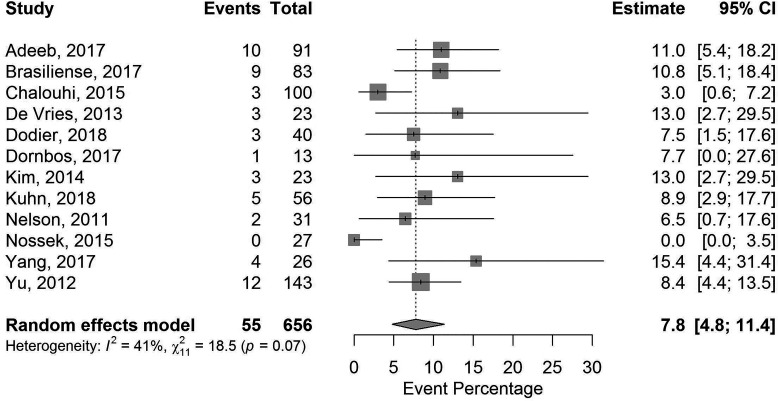
Safety composite endpoint for flow diverters available commercially in the USA.

For the safety analysis, 12 studies reported all components of the composite endpoint and employed only flow diverters commercially available in the USA (figure 2). The overall event rate was 7.8% (95% CI 4.8% to 11.4%). The performance goal, prespecified at the upper bound of the 95% CI, was thus 11.4% for the cumulative occurrence of death, stroke, intracranial hemorrhage and/or mRS worsening. Residual and pre-planned sensitivity analyses found the estimate to be robust. There was no significant difference in the safety composite event between the 12 studies using flow diverters commercially available in the USA and the five studies using flow diverters not commercially available in the USA (p=0.42). There was no evidence of publication bias or small-study effects (online supplement 2). Only two studies in the safety meta-analysis reported use of a clinical events committee or an independent review of safety events.^11 12^

## Discussion

The most important findings from the current meta-analysis for small and medium-sized unruptured aneurysms arising from the intracranial carotid artery include the following: (1) flow diverters are effective in achieving aneurysm exclusion from the circulation with complete occlusion rates of 75%; (2) treatment can be achieved with a rate of safety events of ~8%; and (3) the published effectiveness and safety of devices commercially available in the USA are not different from those available outside the USA.

### Generalizability of the results

The effectiveness and safety data are remarkably consistent across studies and across subtypes of flow diverters, reflecting substantial generalizability for operators in different geographies to achieve similar results using available flow diverters. These results confirm that flow diversion is a reliable and reproducible technique. Physicians should consider these data when considering treatment recommendations for individual patients. Although the rate of safety events is consistent, treatment carries risk, and this risk must be considered in relation to the natural history risk of small cerebral aneurysms.^13^

### Size and locations of aneurysms: implications for effectiveness and safety

The majority of the aneurysms included in the present meta-analysis were <10 mm and arose from the ICA. Of 22 studies (789 patients)with larger mean size (7-12mm), seven studies reported further details on size and only 1/3 of aneurysms were >10 mm. The remaining 15 studies included only the mean/median aneurysm size, and only four (totaling 87 eligible patients) reported this size to be >10 mm. For this reason, we believe that aneurysms >12 mm comprised a small percentage of the aneurysms included in the present study. For ICA location, we required at least 90% of aneurysms to be located in the ICA (or a subgroup of all ICA aneurysms to be extractable). Of all 41 studies, 14 were eligible based on the ≥90% rule (median, 93% ICA). The number of aneurysms in these studies was 1149, so probably fewer than 100 patients with non-ICA aneurysms were included in the present study.

The effectiveness and safety data for the current population of small and medium-sized carotid aneurysms included in the current meta-analysis were generally analogous to the metrics reported in individual, high quality trials, some of which included larger aneurysms. For example, in the Pipeline for Uncoilable and Failed aneurysmS (PUFS) trial, the 1 year occlusion rate reported was 73.6% at 180 days with a progressive increase in the occlusion rate every year through 5 years.^14^ In a pooled analysis of three large studies, which included primarily large (mean size 12 mm) wide-necked (mean neck size 6.6 mm) carotid aneurysms treated with the Pipeline Embolization Device, the authors reported a 75% rate of complete occlusion at 180 days (which again increased over time).^15^

Previous meta-analyses have reported on more heterogeneous groups of studies including both unruptured and ruptured aneurysms of a variety of sizes and anatomical locations. As such, the resultant estimates of safety vary widely between analyses. Many of these prior meta-analysis studies are not directly comparable to the present study which included a highly selected population. For example, Zhou *et al* conducted a meta-analysis including 60 studies with data on 3125 patients treated with either the Pipeline or Silk devices for both ruptured and unruptured aneurysms.^16^ Not unexpectedly, they reported overall complication rates (17%) and mortality rates (2.8%) which greatly exceeded our observations.

One point of consistency seems to be a correlation between higher rates of morbidity and mortality with large and giant aneurysm size. Both Zhou *et al* and Bhatia *et al* observed this relationship.^16 17^ Because our study was limited to small and medium sized aneurysms, no such comparison could be made.

### Limitations

Our study is limited by the fact that certain aneurysm locations, sizes, and parent vessel configurations come with different risk and occlusion profiles. Another important limitation of this meta-analysis is that in many studies the definition of stroke was not made explicit. In general, the composite safety outcomes had to rely on the definitions of events used by the authors of each study, which were often unclear or not fully reported.

Future structural and technical iterations should be based on studies of the minority of aneurysms that fail to occlude after flow diversion and those cases in which complications are more likely to be encountered. The development of lower profile delivery systems, anti-thrombotic coatings, and modified braiding structures represent potential mechanisms for future improvement.

The present meta-analysis provides a robust benchmark on which performance goals can be constructed to evaluate new flow-diversion systems for use in small to medium-sized unruptured carotid aneurysms.
