# Supplementary material for: How safe and effective are flow diverters for the treatment of unruptured small/medium intracranial aneurysms of the internal carotid artery? Meta-analysis for evidence-based performance goals
Source: J Neurointerv Surg. 2020 Jan 31;12(9):869–73. doi: 10.1136/neurintsurg-2019-015535 (PMC7476367; doi:10.1136/neurintsurg-2019-015535)
Supplement: Supplementary data [file neurintsurg-2019-015535supp001.pdf]

SUPPLEMENT 1. Search methods

A systematic search was conducted in the setting of endovascular treatment of intracranial aneurysms for English language studies published between January 1, 2008 and June 30, 2018 or presented at recent professional meetings.

The primary electronic search was conducted in PubMed, the National Library of Medicine's online literature database, with the following search strategy, where "[MeSH]" indicates a Medical Subject Heading and "[tiab]" indicates a keyword to be sought in the title and abstract:

1. Intracranial Aneurysm[MeSH]
2. ("cerebral aneurysm" OR "intracranial aneurysm" OR "unruptured aneurysm" OR "brain aneurysm" OR "cerebral aneurysms" OR "intracranial aneurysms" OR "unruptured aneurysms" OR "brain aneurysms") NOT medline[sb]
3. #1 OR #2
4. "flow diverter" OR "flow diverters" OR "flow diversion" OR "flow-diverting" OR P64 OR Pipeline OR PED[tiab] OR PFED[tiab] OR SILK OR "flow redirection endoluminal device" OR FRED[tiab] OR Surpass[tiab] OR Derivo OR Tubridge
5. #3 AND #4; Limits: English, 2008–2018

In addition to the PubMed search, abstracts presented at specified meetings (International Stroke Conference and Society of NeuroInterventional Surgery) were searched for 2017 and 2018. Manual bibliography checks of recent literature reviews also supplemented the electronic searches.
