# Supplementary material for: How safe and effective are flow diverters for the treatment of unruptured small/medium intracranial aneurysms of the internal carotid artery? Meta-analysis for evidence-based performance goals
Source: J Neurointerv Surg. 2020 Jan 31;12(9):869–73. doi: 10.1136/neurintsurg-2019-015535 (PMC7476367; doi:10.1136/neurintsurg-2019-015535)

SUPPLEMENT 2. Funnel plots and regression tests of publication bias

A. Funnel Plot: Complete Occlusion at 12 Months, Flow Diverters Commercially Available in US

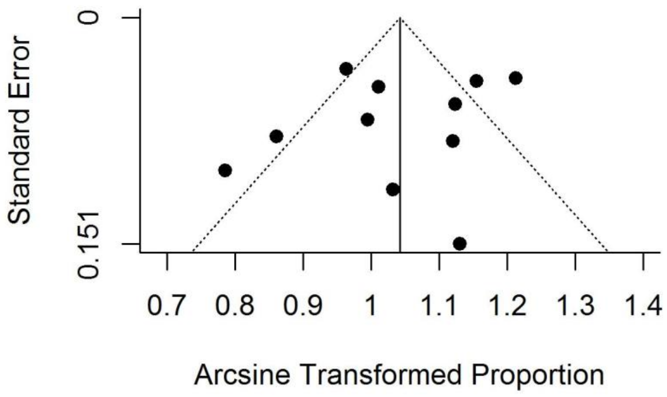

Egger's regression test for funnel plot asymmetry: P=0.4384. There is no evidence of publication bias or small-study effects.

B. Funnel Plot: Complete Occlusion at 12 Months, All Flow Diverters

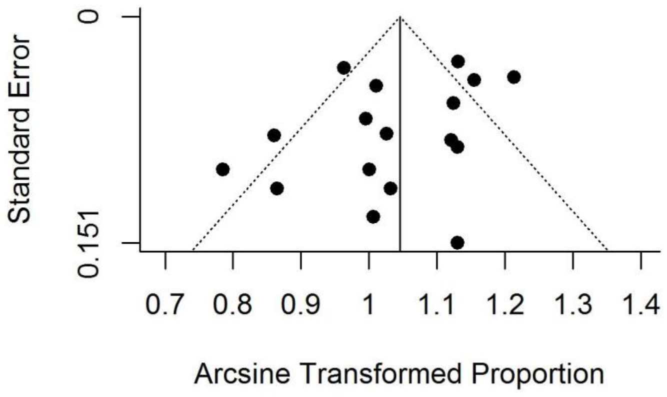

Egger's regression test for funnel plot asymmetry: P=0.1315. There is no evidence of publication bias or small-study effects.

C. Funnel Plot: Safety Composite Endpoint, Flow Diverters Commercially Available in US

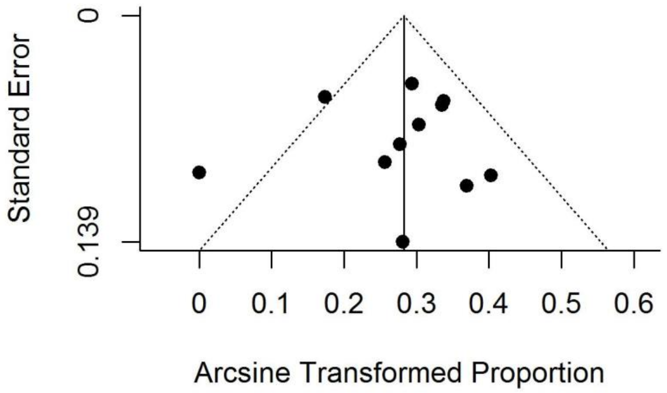

Egger's regression test for funnel plot asymmetry: P=0.9573. There is no evidence of publication bias or small-study effects.

D. Funnel Plot: Safety Composite Endpoint, All Flow Diverters

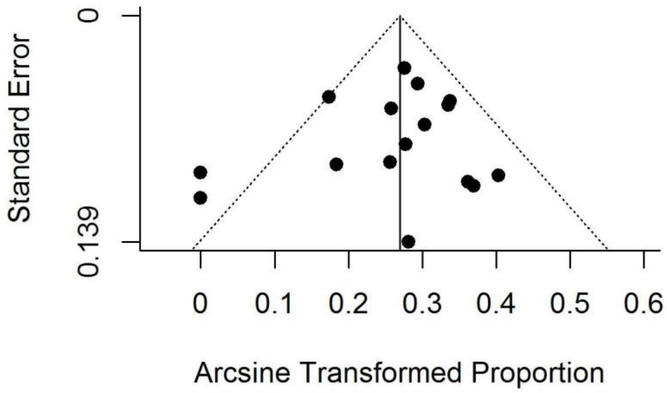

Egger's regression test for funnel plot asymmetry: p = 0.6276. There is no evidence of publication bias or small-study effects.

E. Safety Composite Endpoint, All Flow Diverters

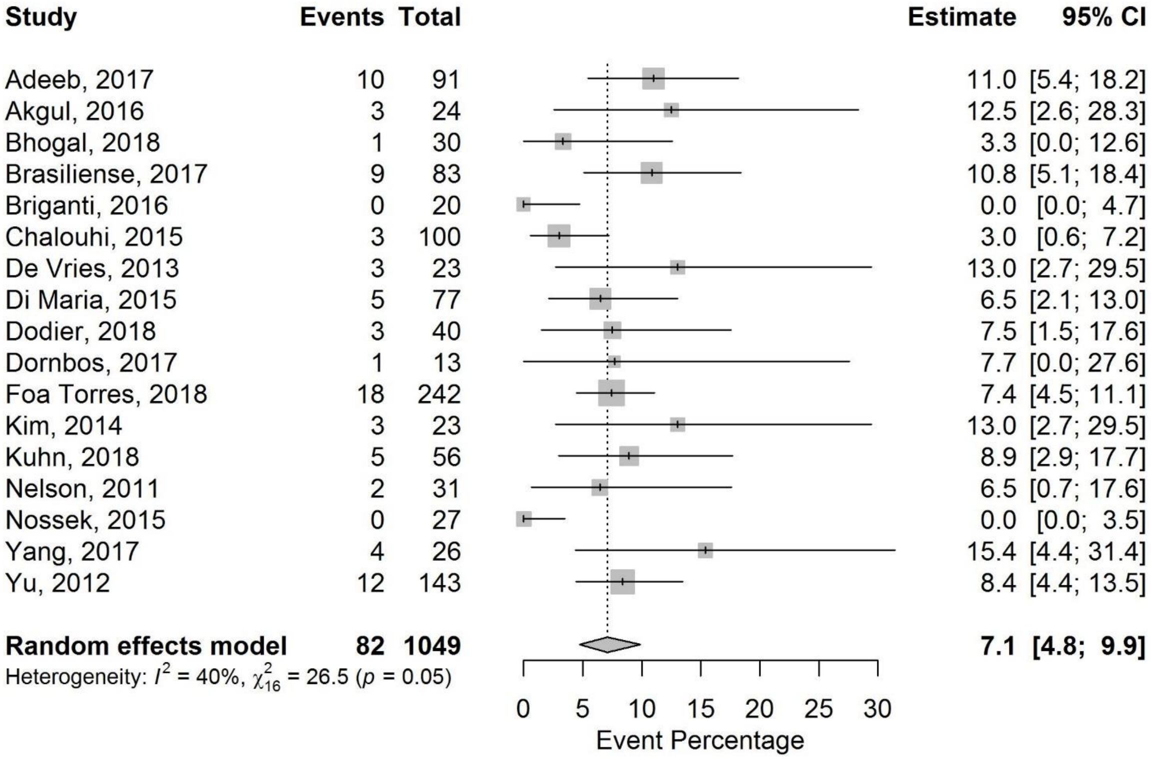

Supplement: Supplementary data [file neurintsurg-2019-015535supp002.pdf]
